# Ceftolozane/tazobactam heteroresistance in cystic fibrosis-related *Pseudomonas aeruginosa* infections

**DOI:** 10.1093/jacamr/dlad083

**Published:** 2023-07-11

**Authors:** Marguerite L Monogue, James M Sanders, Christine A Pybus, Jiwoong Kim, Xiaowei Zhan, Andrew E Clark, David E Greenberg

**Affiliations:** Department of Pharmacy, University of Texas Southwestern Medical Center, Dallas, TX 75390, USA; Department of Internal Medicine, Infectious Diseases and Geographic Medicine, University of Texas Southwestern Medical Center, Dallas, TX 75390, USA; Department of Pharmacy, University of Texas Southwestern Medical Center, Dallas, TX 75390, USA; Department of Internal Medicine, Infectious Diseases and Geographic Medicine, University of Texas Southwestern Medical Center, Dallas, TX 75390, USA; Department of Microbiology, University of Texas Southwestern Medical Center, Dallas, TX 75390, USA; Department of Population and Data Sciences, Quantitative Biomedical Research Center, University of Texas Southwestern Medical Center, Dallas, TX 75390, USA; Department of Population and Data Sciences, Quantitative Biomedical Research Center, University of Texas Southwestern Medical Center, Dallas, TX 75390, USA; Department of Pathology, University of Texas Southwestern Medical Center, Dallas, TX 75390, USA; Department of Internal Medicine, Infectious Diseases and Geographic Medicine, University of Texas Southwestern Medical Center, Dallas, TX 75390, USA; Department of Microbiology, University of Texas Southwestern Medical Center, Dallas, TX 75390, USA

## Abstract

**Objectives:**

Cystic fibrosis (CF) patients are often colonized with *Pseudomonas aeruginosa*. During treatment, *P. aeruginosa* can develop subpopulations exhibiting variable *in vitro* antimicrobial (ABX) susceptibility patterns. Heteroresistance (HR) may underlie reported discrepancies between *in vitro* susceptibility results and clinical responses to various ABXs. Here, we sought to examine the presence and nature of *P. aeruginosa* polyclonal HR (PHR) and monoclonal HR (MHR) to ceftolozane/tazobactam in isolates originating from CF pulmonary exacerbations.

**Methods:**

This was a single-centre, non-controlled study. Two hundred and forty-six *P. aeruginosa* isolates from 26 adult CF patients were included. PHR was defined as the presence of different ceftolozane/tazobactam minimum inhibitory concentration (MIC) values among *P. aeruginosa* isolates originating from a single patient specimen. Population analysis profiles (PAPs) were performed to assess the presence of MHR, defined as ≥4-fold change in the ceftolozane/tazobactam MIC from a single *P. aeruginosa* colony.

**Results:**

Sixteen of 26 patient specimens (62%) contained PHR *P. aeruginosa* populations. Of these 16 patients, 6 (23%) had specimens in which PHR *P. aeruginosa* isolates exhibited ceftolozane/tazobactam MICs with categorical differences (i.e. susceptible versus resistant) compared to results reported as part of routine care. One isolate, PSA 1311, demonstrated MHR. Canonical ceftolozane/tazobactam resistance genes were not found in the MHR isolates (MHR PSA 1311 or PHR PSA 6130).

**Conclusions:**

Ceftolozane/tazobactam PHR exists among *P. aeruginosa* isolates in this work, and approximately a quarter of these populations contained isolates with ceftolozane/tazobactam susceptibiilty interpretations different from what was reported clinically, supporting concerns surrounding the utility of traditional susceptibility testing methodology in the setting of CF specimens. Genome sequencing of isolates with acquired MHR to ceftolozane/tazobactam revealed variants of unknown significance. Future work will be centred on determining the significance of these mutations to better understand these data in clinical context.

## Introduction

Antimicrobial resistance is a global phenomenon that causes significant infection-related morbidity and mortality.^1^ It is imperative that antimicrobials are used appropriately and effectively to optimize patient care and delay the emergence of antimicrobial resistance. Clinically, antimicrobial utilization is guided by antimicrobial susceptibility testing (AST). Although rapid and convenient, the results of AST from complex specimens may misinform treatment decisions in certain situations as a single, representative colony of each morphotype is chosen for work-up. Furthermore, selection of predominating morphotypes may not accurately represent the full compendium of a species present in microbially diverse specimens. Additionally, colonization with different strains of the same species with different susceptibility profiles can occur. In this setting, such organisms may appear morphologically similar in culture, causing resistant subpopulations or heteroresistance (HR) to go undetected.

While the clinical relevance of HR subpopulations is still being established, HR may underlie reported discrepancies between AST data and clinical response. As such, routine AST methodologies (as currently operationalized in clinical microbiology laboratories) may not provide the optimal method to guide interventions in certain settings.^2–7^ Previous reports have identified HR mechanisms among diverse bacterial species and patient populations.^4,8,9^ Cystic fibrosis (CF) patients are often chronically colonized with recalcitrant respiratory pathogens resulting in complex cultures that often contain multiple species. Furthermore, species such as *Pseudomonas aeruginosa* can colonize the host with multiple unique strains, or develop subpopulations that are morphologically and/or genetically similar but exhibit variable antimicrobial susceptibility patterns when tested *in vitro*.^10–12^

In response to increasing levels of resistance and diversification of resistance mechanisms among Gram-negative bacteria (including *P. aeruginosa*), several novel β-lactam/β-lactamase inhibitor combinations have been developed, including ceftolozane/tazobactam.^13^ These agents provide activity against MDR *P. aeruginosa* and are attractive options for management of CF exacerbations. HR to carbapenems (imipenem and meropenem), cephalosporins (cefepime, cefiderocol) and colistin has been well described previously in *P. aeruginosa*, with upwards of 100% of isolates demonstrating HR.^14–18^ However, the prevalence of *P. aeruginosa* HR from CF isolates to first-line anti-pseudomonal agents or novel β-lactam/β-lactamase inhibitors remains poorly described. Identifying the prevalence of HR among CF isolates furthers our understanding of the limitations associated with routine AST methods and betters our understanding of how HR is related to patient outcomes. This study aimed to interrogate the presence and nature of polyclonal and monoclonal HR (PHR and MHR, respectively) to ceftolozane/tazobactam in *P. aeruginosa* isolates originating during CF pulmonary exacerbations. The roles of prior antimicrobial exposure and synergistic antimicrobial regimens in relation to the development of *P. aeruginosa* HR to ceftolozane/tazobactam were evaluated.

## Materials and methods

### Antimicrobial agents

Ceftolozane (Lot# 44042000041) and tazobactam (Lot# 5/08) analytical powders were obtained from Merck & Co., Inc., NJ, USA. Ceftolozane and tazobactam were reconstituted using DMSO. Subsequent dilutions in Mueller–Hinton (MH) broth were made to attain final concentrations for the population analysis profile (PAP) analyses. Commercial vials of amikacin (Lot# VEAC040) and ciprofloxacin (Lot# A0E0423A) were used in the time–kill analyses.

### Isolates and antimicrobial susceptibility studies

This was a single-centre, non-controlled study. Sputum cultures from 26 adult CF patients admitted from January 2018 to August 2020 to Clements University Hospital (CUH) were collected consecutively. The clinical details concerning the reason for admission and sputum culture were not recorded.

Sputum cultures were ordered for each enrolled patient and were processed, and results reported according to established laboratory methodology as part of routine care. For this work, 5 to 10 additional *P. aeruginosa* colonies with similar morphotypes were selected, isolated and added to the UTSW Texas Infectious Diseases Biorepository (IRB-approved protocol STU-2018-0319). The identities of all isolates as *P. aeruginosa* were verified by MALDI-TOF MS (Bruker, Billerica, MA, USA). To revive cultures from cryostorage at −80°C, each isolate was subcultured twice on trypticase soy agar with 5% sheep blood prior to use.

Susceptibility testing in this work was performed by both broth microdilution and gradient diffusion. Antibiotic strip gradient diffusion (Fisher Scientific Company) was conducted for ceftolozane/tazobactam (Lot# 100120008), ceftazidime/avibactam (Lot# 052620020) and imipenem/relebactam (Lot# 100520063). AST results were interpreted using criteria as described in the CLSI M-100 document.^19^ MIC experiments were repeated in triplicate in isolates exhibiting phenotypic inconsistencies across AST results.

### PHR

Ceftolozane/tazobactam susceptibility testing was performed as described above for each individual colony from the 5–10 stored *P. aeruginosa* isolates (per patient) to detect phenotypically mixed populations. PHR is defined as the presence of different susceptibility profiles among the colonies that originated from a single CF sputum specimen. PHR was determined to be present when *P. aeruginosa* culture isolates met one of the following criteria: (i) ≥2-fold MIC change; (ii) >2-fold MIC change; and/or (iii) susceptibility interpretation MIC change.^8,20^ Isolates that exhibited HR were genome sequenced to determine genetic relatedness and evaluate the presence of canonical antibiotic resistance mechanisms.

### MHR

Of the patient cultures that demonstrated PHR, PAPs for ceftolozane/tazobactam were conducted for a minimum of two *P. aeruginosa* isolates within the same patient specimen. Isolates were selected based on the following criteria: (i) the two isolates that demonstrated the greatest variation in MIC; and/or (ii) isolates with HR subpopulations within the zone of inhibition on antibiotic strip gradient diffusion. MHR was defined as organism growth on PAP at ≥4-fold the MIC. Isolates were screened for MHR with modified PAPs at concentrations 2- and 4-fold the ceftolozane/tazobactam MIC (e.g. if the ceftolozane/tazobactam MIC was 1/4 mg/L, PAPs were conducted at 4/4 and 16/4 mg/L). MH agar plates were supplemented with concentrations of ceftolozane reflective of the fold increase from the original ceftolozane/tazobactam MIC. The tazobactam concentration was held at 4 mg/L. From an overnight culture of the test isolate, an estimated 10^8^ cfu were spread on each plate. Organisms that grew on plates with 4-fold the ceftolozane/tazobactam MIC were repeated in triplicate on complete PAPs at 2-, 4-, 6- and 8-fold the ceftolozane/tazobactam MIC.

For complete PAPs, an identical culture volume was serially diluted and plated on MH agar without antibiotic selection to quantitate the actual number of cfu plated. Colonies were counted after 24–48 h of growth at 37°C. The number of resistant isolates at each concentration was determined as a percentage of the total number of inoculated cfu to determine the proportion of cells within the initial culture that exhibited MHR.

### Time–kill studies: HR induction and prevention

To determine the impact on preventing or reverting ceftolozane/tazobactam HR, time–kill analyses were performed on *P. aeruginosa* isolates with confirmed MHR to ceftolozane/tazobactam. The initial ceftolozane/tazobactam-susceptible and post-PAP ceftolozane/tazobactam-resistant isolates were preferentially selected. Antimicrobial exposures were based on estimated free trough concentrations of ceftolozane/tazobactam (8/2 mg/L) and common non-β-lactam antimicrobials used in CF exacerbations, amikacin (2 mg/L) and ciprofloxacin (0.5 mg/L) were tested.^21^ Each isolate was subcultured twice on trypticase soy agar with 5% sheep blood (Becton, Dickinson & Co., Sparks, MD, USA). MH broth was inoculated with the bacterial suspension of ∼1 × 10^8^ cfu/mL in 5 mL, colourless culture tubes (Falcon, Fisher Scientific). Control experiments without active compound were included. Singular studies were conducted unless initial bacterial log growth across arms was inconsistent. Final volumes for each *P. aeruginosa*–drug concentration were 5 mL and incubated at 37°C using a shaker at 250 rpm. Samples were taken from each culture tube at 0, 3, 6 and 24 h from the time of broth inoculation. Multiple 1:10 dilutions were made in saline and subcultured onto blood agar plates. Plates were incubated for 18–24 h at 37 °C and mean bacterial densities in cfu/mL were determined for each isolate.

### Genome sequencing

Genome sequencing was conducted utilizing Illumina Miseq. If available, two isolates with different ceftolozane/tazobactam MICs (≥2-fold) were selected for sequencing from PHR and MHR populations. DNA was isolated using the ZymoBIOMICS DNA miniprep kit (Zymo Research, Irvine, CA, USA) and library preparation and sequencing was performed by the UTSW Sequencing Core. PE150 runs were performed to a depth of 40 ×  coverage. Data were analysed using algorithms, as described previously, to determine genetic mechanisms of resistance.^22,23^ Trim Galore (https://www.bioinformatics.babraham.ac.uk/projects/trim_galore/) was used for quality and adapter trimming. SPAdes (v3.14.0) was used for *de novo* genome assembly and MUMmer 4 was used to compare the genome assemblies.^24,25^ The mutation rate between two genomes was calculated by the number of SNPs divided by the alignment length and Jukes–Cantor distance was calculated from the mutation rate. The neighbour-joining tree was generated based on the distances using R packages ape and ggtree.^26,27^ The ST was defined by mapping the sequencing reads to the allele sequences from PubMLST (https://pubmlst.org) using Burrows–Wheeler Aligner (BWA, v0.7.17).^28^

### Determination of antimicrobial exposure

Patients from whom *P. aeruginosa* isolates utilized in this work originated were retrospectively reviewed for type (IV, oral) and duration of antimicrobial exposure prior to culture collection. The total amount of antimicrobial exposure is described as days of therapy. Differences in total antimicrobial exposure and ceftolozane/tazobactam exposure between patients demonstrating PHR and those who did not were explored via an unpaired *t*-test (2022 GraphPad software). A *P* value of ≤0.05 was considered significant.

## Results

### Antimicrobial susceptibility studies

**Figure 1.**
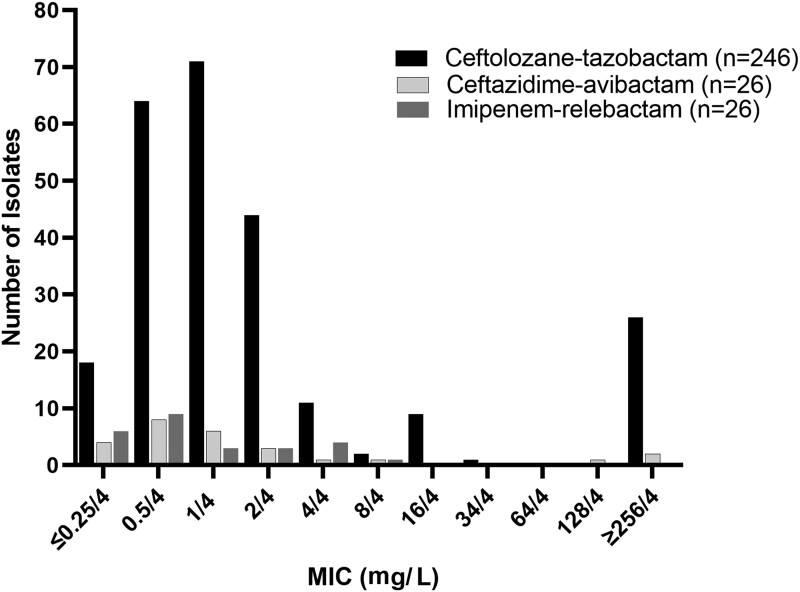
Ceftolozane/tazobactam, ceftazidime/avibactam and imipenem/relebactam MIC distributions against *P. aeruginosa* isolates from 26 adult CF patients.

Two hundred and forty-six *P. aeruginosa* isolates from 26 unique adult CF patients were tested against ceftolozane/tazobactam. The ceftolozane/tazobactam MICs ranged from ≤0.25/4 to ≥256/4 mg/L. The ceftolozane/tazobactam MIC_50_ and MIC_90_ were 1/4 and ≥256/4 mg/L, respectively (Figure 1). The first isolate from each patient (*n* = 26) also underwent ceftazidime/avibactam and imipenem/relebactam susceptibility testing. The ceftazidime/avibactam MICs ranged from ≤0.25/4 to ≥256/4 mg/L, with the MIC_50_ and MIC_90_ determined to be 1/4 and 128/4 mg/L, respectively. The imipenem/relebactam MICs ranged from ≤0.25/4 to 8/4 mg/L, with the MIC_50_ and MIC_90_ determined as 0.5/4 and 4/4 mg/L, respectively. Of the 4 ceftolozane/tazobactam-resistant isolates from the original 26 colonies, all 4 isolates were ceftazidime/avibactam resistant, with varying susceptibility to imipenem/relebactam (1 susceptible, 2 intermediate and 1 resistant).

### PHR

**Figure 2.**
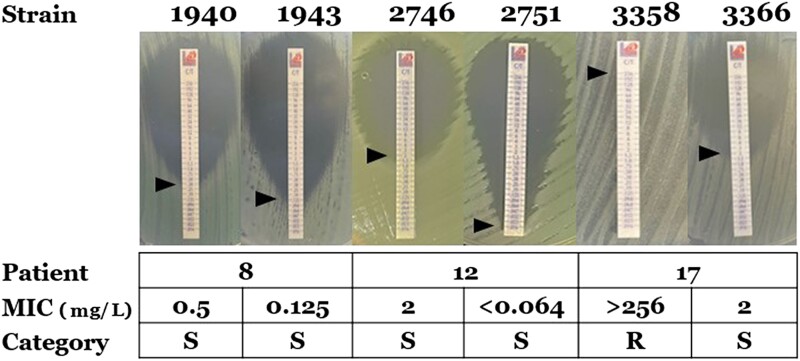
Examples of isolates demonstrating PHR. Isolates 1940 (MIC 0.5 mg/L) and 1943 (MIC 0.125 mg/L) demonstrate 2-fold change in ceftolozane/tazobactam MIC (Patient 8). Isolates 2746 (MIC 2 mg/L) and 2751 (MIC <0.064 mg/L) demonstrate >2-fold change in ceftolozane/tazobactam MIC (Patient 12). Isolates 3358 (MIC >256 mg/L) and 3366 (MIC 2 mg/L) demonstrate a change in susceptibility interpretation. S, susceptible; R, resistant.

Sixteen of the 26 patients (62%) demonstrated a ≥2-fold change in ceftolozane/tazobactam MIC between isolates from the same respiratory culture. Of these 16 patients, the fold change in ceftolozane/tazobactam MIC was >2-fold for seven isolates (27%) and resulted in a change in susceptibility categorical interpretation for six of the isolates (23%). Examples of these PHR isolates are shown in Figure 2. Eight isolates from five unique patients demonstrated growth of HR colonies within the zone of inhibition in gradient diffusion experiments.

### MHR

**Figure 3.**
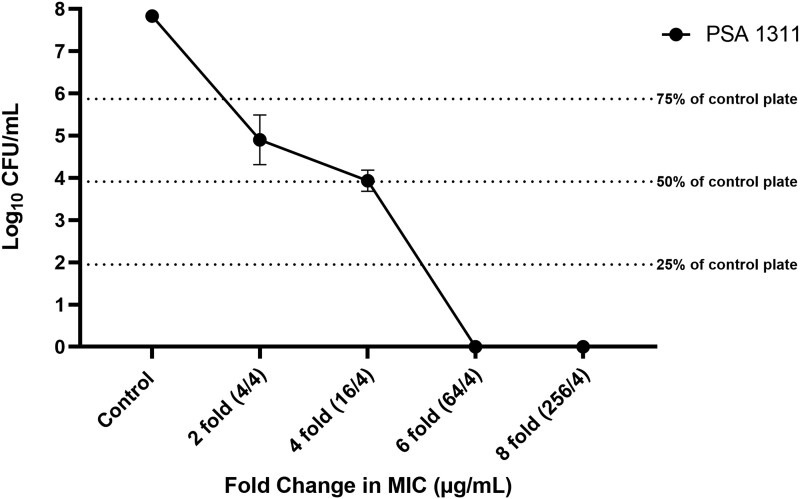
Results from PSA 1311 (ceftolozane/tazobactam MIC 1/4 mg/L) PAPs at 2-, 4-, 6- and 8-fold the ceftolozane/tazobactam MIC. PSA 1311 demonstrated growth on PAPs up to 4-fold the MIC (16/4 mg/L). No growth was seen at 6- or 8-fold the ceftolozane/tazobactam MIC. The isolate recovered at 4-fold ceftolozane/tazobactam MIC was designated PSA 6130 and saved for additional analyses.

Thirty-two *P. aeruginosa* isolates underwent modified PAP analysis. Of these, 28 isolates were ceftolozane/tazobactam susceptible (MIC ≤ 4 mg/L). Of the 32 isolates, 7 grew on MH plates at 2-fold the ceftolozane/tazobactam MIC concentration. Two isolates grew on MH plates at 4-fold the ceftolozane/tazobactam MIC concentration. Of these two isolates, only PSA 1311 was initially ceftolozane/tazobactam susceptible and underwent further PAP analyses. PSA 1311 demonstrated growth on PAPs up to 4-fold the MIC (16/4 mg/L) (Figure 3). No growth was seen at 6- or 8-fold the ceftolozane/tazobactam MIC. The isolate recovered at 0-fold (no drug) and 4-fold ceftolozane/tazobactam MIC were designated as PSA 6129 and PSA 6130, respectively, and saved for additional analyses. The incidence of ceftolozane/tazobactam *P. aeruginosa* MHR in ceftolozane/tazobactam susceptible isolates was 3.6%.

### Time–kill studies: HR induction and prevention

**Figure 4.**
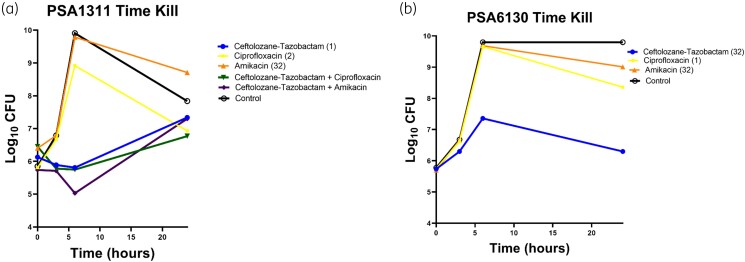
Time–kill experiments displaying the activity of antimicrobials alone and in combination at trough concentrations against (a) PSA 1311 and (b) PSA 6130 (MIC). Against PSA 1311 (ceftolozane/tazobactam MIC 1 mg/L), bacterial regrowth from baseline was observed by 24 h. No reduction in bacterial density over time was observed with ciprofloxacin (MIC 2 mg/L) and amikacin (MIC 32 mg/L) monotherapy. At 6 h, the combinations of amikacin plus ceftolozane/tazobactam and ciprofloxacin plus ceftolozane/tazobactam reduced the bacterial density, but regrowth above baseline was observed by 24 h. Against PSA 6130 (ceftolozane/tazobactam MIC 32 mg/L), there were no reductions in bacterial density in the ceftolozane/tazobactam, ciprofloxacin (MIC 1 mg/L) and amikacin (MIC 32 mg/L) arms.

Against PSA 1311 (ceftolozane/tazobactam MIC 1 mg/L), the change in bacterial density from 0 h for ceftolozane/tazobactam was −0.32 log_10_ cfu at 6 h, but regrowth up to +1.21 log_10_ cfu from baseline was observed by 24 h. No reduction in bacterial density over time was observed with ciprofloxacin (MIC 2 mg/L) and amikacin (MIC 32 mg/L) monotherapy. At 6 h, the combinations of amikacin plus ceftolozane/tazobactam and ciprofloxacin plus ceftolozane/tazobactam reduced the bacterial density from 0 h by −0.71 and −0.70 log_10_ cfu, respectively, but regrowth above baseline was observed by 24 h. Against PSA 6130 (ceftolozane/tazobactam MIC 32 mg/L), there were no reductions in bacterial density in the ceftolozane/tazobactam, ciprofloxacin (MIC 1 mg/L) and amikacin (MIC 32 mg/L) arms (Figure 4).

### Genome sequencing

**Figure 5.**
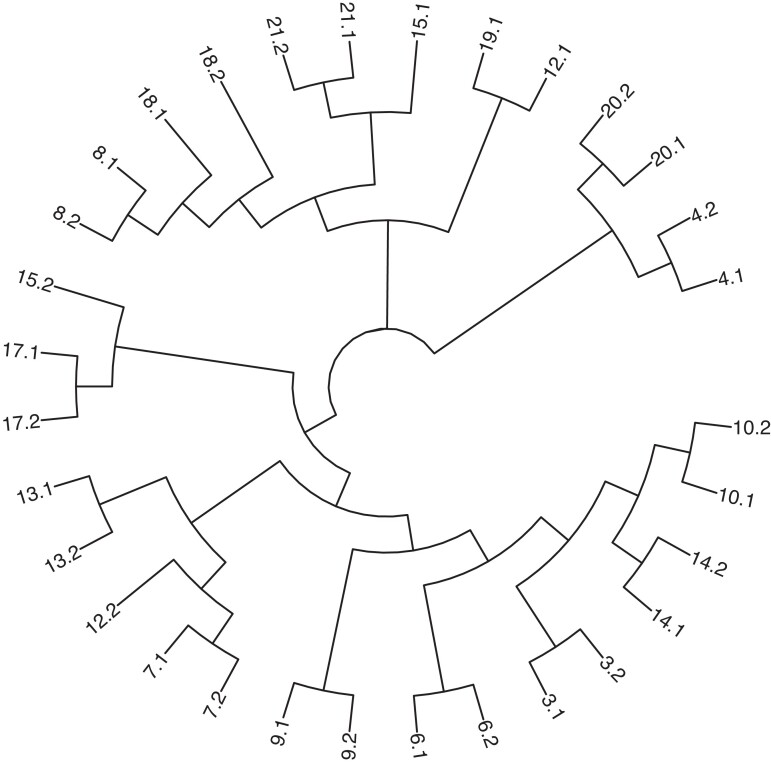
Phylogenetic relationships between isolates (labelled ‘patient number.sample number’) displaying ceftolozane/tazobactam HR. HR *P. aeruginosa* isolates from CF respiratory specimens exhibit a high level of clonality.

A total of 31 isolates from 16 patients were sequenced. Isolate 19.2 was not available for sequencing. PSA 1311 and 6130 were determined to be ST776 by MLST analysis (100%) with a mutation rate of 0.00004% and SNP difference of 232 between the two isolates. Known ceftolozane/tazobactam resistance genes were not found in PSA 1311 and 6130; however, hypothetical protein variants were identified. Phylogenetic relationships between HR isolates are displayed in Figure 5. Each patient’s isolates were represented by a unique MLST. Despite a given patient’s isolates being the same ST, they were not clonal and had numerous SNPs that were different between the HR isolates.

### Determination of antimicrobial exposure

Total antimicrobial exposure (354 versus 419 days, *P* > 0.05) and ceftolozane/tazobactam exposure (3.7 versus 3.8 days, *P* > 0.05) did not differ from patients demonstrating PHR versus those that did not. Patient 6 (isolates PSA 1311 and PSA 6130) did not have any documented ceftolozane/tazobactam exposure.

## Discussion

CF patients are often colonized with multiple strains of *P. aeruginosa*. *P. aeruginosa* can also develop subpopulations exhibiting variable *in vitro* antimicrobial susceptibility patterns. HR may underlie discrepancies between results of *in vitro* susceptibility testing and clinical responses to various antimicrobials.^4,17^ Here, we sought to examine the presence and nature of *P. aeruginosa* HR to ceftolozane/tazobactam in isolates originating from CF patients.

Susceptibilities to ceftolozane/tazobactam and ceftazidime/avibactam were similar across our CF *P. aeruginosa* isolates. Compared with ceftolozane/tazobactam and ceftazidime/avibactam, imipenem/relebactam exhibited better *in vitro* potency. Most of the CF isolates were susceptible to ceftolozane/tazobactam; however, a large portion (>20%) exhibited high-level resistance with MICs of ≥256 mg/L. To our knowledge, this is the first study comparing the activity of these three novel agents against adult CF-specific respiratory isolates. Previous work has shown that the MIC_50_/MIC_90_ values of ceftolozane/tazobactam and ceftazidime/avibactam were 0.5/2 and 2/8 mg/L, respectively, when examining a collection of 6839 *P. aeruginosa* isolates (non-CF or respiratory specific).^29^ In a study comparing ceftolozane/tazobactam, ceftazidime/avibactam and imipenem/relebactam against non-respiratory *P. aeruginosa* isolates, all three agents were active against 98% of isolates.^30^ In comparison, our CF-specific data raise questions surrounding the future utility of this agent for the treatment of CF exacerbations as resistance development is a concern.

Our findings demonstrate that ceftolozane/tazobactam PHR exists among *P. aeruginosa* isolates in most of the CF patients examined in this work. The presence of roughly a quarter of these PHR isolates resulted in categorical changes in susceptibility. PHR has been described in CF patients, with an incidence of 64% against 11 different antimicrobials, which was much greater than in non-CF patients.^10^ In addition, clinically undetected resistant subpopulations of *P. aeruginosa* are frequently found in CF sputum samples.^11^ These data further support concerns surrounding the clinical utility of routine susceptibility testing methodologies for CF specimens.

Our data suggest MHR also exists, albeit less frequently than PHR, in our small patient cohort. Canonically, two definitions have been used to define HR: (i) growth of a resistant subpopulation at ≥2-fold the susceptibility breakpoint at a frequency of ≥0.0001% (1 × 10^−6^) from the original population; and (ii) growth of a resistant subpopulation at antimicrobial concentration ≥8-fold the MIC of the main population, at a frequency of ≥0.00001 (1 × 10^−7^) from the original population.^4,5,8^ Our HR subpopulation meets the former definition. The ceftolozane/tazobactam MIC of the original isolate (PSA 1311) was 1/4 mg/L and the clinical breakpoint is 4/4 mg/L. The HR subpopulation demonstrated growth up to an MIC of 16/4 mg/L (2-fold the breakpoint and 4-fold the MIC) and the subpopulation represented 0.01% of the original population. The incidence of ceftolozane/tazobactam *P. aeruginosa* MHR in ceftolozane/tazobactam-susceptible isolates was 3.6%.

In comparison with prior studies of other antimicrobials, the frequency of ceftolozane/tazobactam MHR in *P. aeruginosa* was much lower than what has been described with colistin, carbapenems and cefiderocol. Colistin MHR in *P. aeruginosa* ranged from 4.2% to 37.5%, although definitions of MHR in these studies were broader.^31,32^ Carbapenem MHR in *P. aeruginosa* was 28.5%, but these isolates were preferentially selected based on colony growth within the zone of inhibition.^14^ Cefiderocol MHR in *P. aeruginosa* was detected in 7% of cefiderocol-susceptible isolates.^18^ Thus, while our population was small, ceftolozane/tazobactam MHR appears to be less of a concern in comparison with other anti-*P. aeruginosa* antimicrobials.

Among MHR isolates, neither the presence of plasmids nor previously characterized antibiotic resistance mutations could explain the vast change in ceftolozane/tazobactam MICs between PSA 1311 and PSA 6130 in this work. MLST mapping to PSA ST776 for both isolates demonstrated close intrahost relatedness with a mutation rate of 0.00004%. The lack of an obvious cause of HR has been described previously in CF patients.^10^ Additional data are needed to determine the significance of the genetic variants that exist between these strains.

Interestingly, the MHR subpopulation (PSA 6130) produced an MIC of ≥256 mg/L by gradient diffusion when derived directly from the PAP plate (16/4 mg/L), but notably no growth was observed beyond concentrations of 16/4 mg/L on the PAPs. The resistance of PSA 6130 remained stable after 10 passes on non-antibiotic-containing blood agar plates, similar to what was observed with carbapenems and *P. aeruginosa*.^14^ The discrepancy between PAP growth (16/4 mg/L) and gradient diffusion (≥256 mg/L) directly from the same isolate remains unclear.

The patient from whom the MHR isolate was derived did not receive ceftolozane/tazobactam; therefore, we were unable to assess the clinical impact ceftolozane/tazobactam exposure had on patient outcomes. We hypothesize the pharmacokinetic/pharmacodynamic exposure would be compromised, resulting in poor clinical outcomes, albeit data surrounding patient outcomes in the setting of HR are inconsistent. Howard-Anderson and colleagues observed the impact that colistin HR had on clinical outcomes in patients with carbapenem-resistant *P. aeruginosa*. They did not observe an association between colistin HR and 30 or 90 day mortality; however, the majority of these patients did not receive colistin, compromising the interpretation of these results.^17^ Total antimicrobial exposure and ceftolozane/tazobactam exposure within the prior 2 years was not associated with PHR. This is not surprising, as prior studies have identified HR prior to the approval of the specific antimicrobial, suggesting other antimicrobial exposures may influence HR development.^18^

The time–kill studies presented here revealed that exposing the pre-MHR isolate to combination therapy did not prevent the development of HR. However, in our study, combination agents were given at relatively low concentrations representative of trough exposures, below the corresponding MIC. Prior studies have shown combination therapy to be an effective strategy in overcoming HR.^33^ This is an important observation that supports current clinical practices of giving two anti-*P. aeruginosa* antimicrobials for the treatment of a CF exacerbation.

Limitations of this study include the small number of CF patients and the lack of ceftolozane/tazobactam exposures clinically, restricting our interpretation of HR’s clinical impact. The analysis of our time–kill study would be more informative with the use of additional antimicrobials at various concentrations. Lastly, we were unable to show mutations in known resistance genes; therefore, we were unable to completely define the cause of HR in our population.

Our data demonstrate how AST performance based upon the predominant morphological appearance of colonies recovered in culture may lack the sensitivity to detect these nuanced, yet diverse populations. In the setting of PHR, resistant clones with colony morphologies that are similar in appearance to susceptible ones may go overlooked. MHR can only be identified in the presence of antimicrobial selection; however, as the frequency of resistant cells is often very small, it can be routinely missed during a routine AST set-up that analyses only the predominant morphotypes present in each clinical sample. Thus, more sensitive methodologies are necessary to detect HR (e.g. PAP, WGS etc.), which are not yet available for routine use due to lack of clinical data/validation, cost, time-intensive nature, turnaround time and availability of supplies and technology. This is the first study identifying both PHR and MHR to a novel β-lactam/β-lactam inhibitor, ceftolozane/tazobactam, in CF *P. aeruginosa* isolates.
